# Stereological Analysis of Neuron, Glial and Endothelial Cell Numbers in the Human Amygdaloid Complex

**DOI:** 10.1371/journal.pone.0038692

**Published:** 2012-06-13

**Authors:** María García-Amado, Lucía Prensa

**Affiliations:** Departamento de Anatomía, Histología y Neurociencia, Facultad de Medicina, Universidad Autónoma de Madrid, Madrid, Spain; Oregon Health & Science University, United States of America

## Abstract

Cell number alterations in the amygdaloid complex (AC) might coincide with neurological and psychiatric pathologies with anxiety imbalances as well as with changes in brain functionality during aging. This stereological study focused on estimating, in samples from 7 control individuals aged 20 to 75 years old, the number and density of neurons, glia and endothelial cells in the entire AC and in its 5 nuclear groups (including the basolateral (BL), corticomedial and central groups), 5 nuclei and 13 nuclear subdivisions. The volume and total cell number in these territories were determined on Nissl-stained sections with the Cavalieri principle and the optical fractionator. The AC mean volume was 956 mm^3^ and mean cell numbers (x10^6^) were: 15.3 neurons, 60 glial cells and 16.8 endothelial cells. The numbers of endothelial cells and neurons were similar in each AC region and were one fourth the number of glial cells. Analysis of the influence of the individuals’ age at death on volume, cell number and density in each of these 24 AC regions suggested that aging does not affect regional size or the amount of glial cells, but that neuron and endothelial cell numbers respectively tended to decrease and increase in territories such as AC or BL. These accurate stereological measures of volume and total cell numbers and densities in the AC of control individuals could serve as appropriate reference values to evaluate subtle alterations in this structure in pathological conditions.

## Introduction

The cellular composition of the amygdaloid complex (AC) basically consists of three cell populations: neurons, glia and endothelial cells. With the exception of endothelial cells, the number, density and morphology of these cell populations in the distinct nuclei of the AC and the possible changes occurring in pathologies such as schizophrenia or autism have been extensively discussed during the last years [1], [2], [3], [4], [5], [6], [7], [8].

A previous investigation has analyzed the neuronal number and density in the various AC nuclei of control individuals [1], and more studies have compared neuronal density and number between control subjects and individuals with schizophrenia or bipolar disorder [2], [3], [4], [5], [6], and autism [7]. Fewer studies have focused on the density or number of glial cells in the AC [3], [4], [6], [8], and only one of these analyzed these aspects in separated AC nuclei [3], while the others estimated glial density in the AC as a whole. The study analyzing separate AC nuclei reported slight differences in the size and density of the glial cells among the nuclei of the basolateral group in the AC [3]. Previous studies that had compared control subjects and patients with major depression reported a reduction in glial density and glia-neuron ratio [4], [8] in the AC, due to a decrease in the number of oligodendrocytes [8].

The endothelial cells of the AC have been considered in one study. It compared microvessel length in the lateral nucleus of the AC between schizophrenic and control subjects, and reported finding no change in this aspect [9]; however, there are no studies focused specifically on the endothelial cells.

In an exhaustive review of the bibliography no studies focused on aging effects on anatomical measures could be found. fMRI studies reported a decrease in the functional connectivity of the AC with age [10] that might be related to a loss of AC neurons. Though there are no investigations focused on analyzing the changes in the amount of AC neurons between young and aged individuals (longevity), a decrease in neocortical neuron number during aging has been reported [11]. Neuron and glia numbers in the basolateral amygdaloid nucleus have been analyzed in recent studies done in rats from preweaning through old age; there was an aged-related increase in glial cell number as well as neuronal dendritic hypertrophy with age [12]–[14].

Understanding of how AC dysfunction may be related to the pathogenesis of human disorders or accompany behavioral impairments requires a profound knowledge of the normal anatomy of the human AC. For this reason, the main goal of this study was to analyze the density and total number of neurons, glia and endothelial cells in the AC of control individuals. Our stereological approach was consequently designed to provide the total cell number in the AC. No previous studies have focused on analyzing all these cells at the same time in the same sample. We aimed at obtaining accurate stereological measurements of these cell types in every nucleus of the AC, including those that have not been previously analyzed in other studies of the AC, such as the medial nucleus of the corticomedial group or the central nucleus. Additionally, we have also investigated here whether the number or density of any of these cell populations or the volume of any nucleus changed with the individuals’ age at death. Although this investigation was not aimed at performing an aging study, such an analysis would provide valuable information about the effect of age on the number and density of cells in the AC of control individuals. To facilitate the use of stereological techniques in future studies, this paper provides a detailed description of the guidelines used to delineate the nuclei and their subdivisions in the human AC.

## Materials and Methods

### Tissue Processing

The postmortem human samples used in this study were obtained from seven adult individuals (four males and three females) of different ages without clinical or pathological evidence of neurological or psychiatric disorders (Table 1). This tissue was kindly provided by two different sources: the Banco de Tejidos Neurológicos de Navarra, Pamplona, Spain and the Servicio de Anatomía Patológica del Hospital Ramón y Cajal, Madrid, Spain. In the latter, at the time of the decease, the relatives of the patients were asked for written authorization to perform the medical autopsy. Then, many medical samples were anonymized and kept in the hospital for research purposes. The biological samples of the present study were provided by these two Institutions after the approval of our specific project by the corresponding Ethical Committees where the samples were taken (Banco de Tejidos Neurológicos de Navarra and Comité Ético de Investigación Clínica del Hospital Ramón y Cajal) and where the project was performed (Comité de Ética de la Investigación de la Universidad Autónoma de Madrid). Brains were cut into 0.5–1 cm thick coronal slices which were fixed by immersion in 4% paraformaldehyde at 4°C for 4–5 days. The slices were immersed in sucrose 15% and 30% in 0.1 M phosphate buffer (PB; pH 7.4) solutions until they sank before cutting on a freezing microtome into 50 µm thick slices; the resulting sections were collected in an antifreeze solution containing 0.05 M PB, pH 7.4, with 30% ethylene glycol and 30% glycerol and stored at −20°C.

**Table 1:** Clinical data on the human postmortem cases used in the study.

Case	Hemisphere	Age (years)	Sex	Postmortem delay (hrs)	Weight (gr) *	Cause of death
1	L	20	Female	2	1100	Cardiac arrest/cystic fibrosis
2	–-	50	Male	5	1250	Epidermoid carcinoma
3	L	50	Male	4	–-	Liver failure/hyperthyroidism and thyroid breakdown
4	L	75	Female	3	1450	Cardiac tamponade
5	L	68	Male	16	1289	Dilated cardiomyopathy
6	R	56	Male	5	1200	Cardio respiratory arrest
7	R	46	Female	7	1165	Bleach and ammonia ingestion/poisoning

* Weight of the unfixed brain.

### Stainings of the Sections

#### Nissl, acetylcholinesterase and gallyas

To reveal the neurons, glia and endothelial cells of the AC, one section every 1.05 mm of the AC sections of each case was selected starting from a random starting point chosen within the first 1.05 mm of the AC sections; this series was processed for Nissl staining [15]. To delineate the boundaries of the AC nuclei and their subdivisions, two sections adjacent to the one stained for Nissl were processed for acetylcholinesterase [16] and with the Gallyas technique for myelin [17]; the specific protocols for the Gallyas and acetylcholinesterase stainings have been described previously [18], [19].

**Figure 1:**
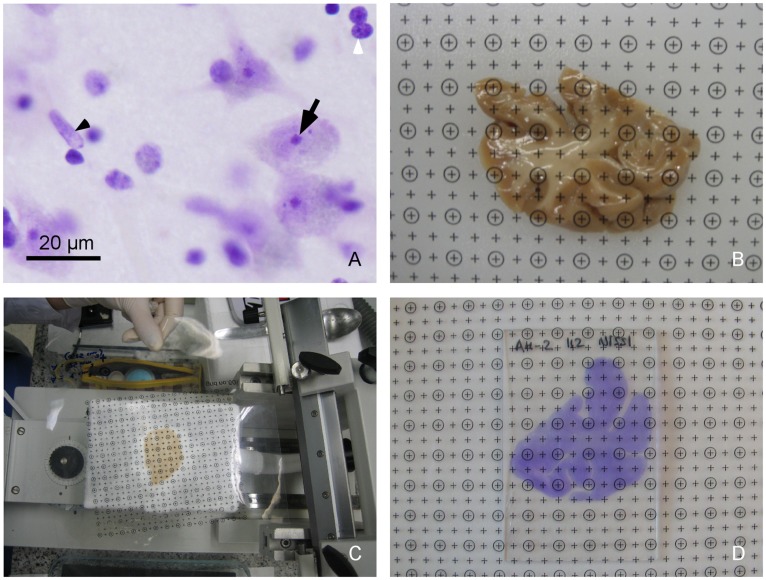
Photomicrographs showing different steps of the stereological procedure. A . Photomicrograph with a 60×oil immersion lens showing different types of cells in the AC. The black arrow points to the nucleolus of one neuron, the white arrowhead points to a glial cell and the black arrowhead points to the nucleus of an endothelial cell. **B**. Grid of points superimposed over the posterior side of a sucrose-immersed tissue block containing the AC. **C**. Grid of points superimposed over the tissue block while it is being cut in the freezing microtome. **D**. Grid of points superimposed over a Nissl-stained section of the tissue block.

Neurons, glia and endothelial cells were identified in the Nissl-stained sections using morphological criteria [20]. Neurons were distinguished from glia on the basis of size, the presence of euchromatin in the nucleus, a clearly visible nucleolus and surrounding cytoplasm (Fig. 1A). Glial cells had heterochromatin in the nucleus and no visible cytoplasm (Fig. 1A). Endothelial cell nuclei were normally part of a blood vessel or had a curved shape (Fig. 1A). As the nucleolus of glial and endothelial cells was not clearly visible in all cases, we considered the equator of the nucleus as the counting unit.

#### NeuN immunohistochemistry

To assure that the identification of the neurons in Nissl-stained sections was correct, another series of sections with a random start from case 7 were treated to reveal the DNA-binding, neuron-specific protein NeuN (neuronal nuclei), contained in the nucleus and the nucleolus of the neuron. The number of neurons identified in the NeuN-stained sections was then counted using the same stereological parameters (such as sampling fractions and counting unit) as in the Nissl countings. The results of these counting are provided in Table S1.

To visualize NeuN, sections were incubated overnight with a monoclonal mouse antibody (Millipore, Billerica, MA; product number MAB377; concentration of 1∶100) and revealed with a biotinylated secondary antibody made in horse (Vector Labs, Burlingame, CA; product number BA2000; concentration 1∶250). NeuN antibody recognized 2–3 bands in the 46–48 kDa range and possibly another band at approximately 66 kDa on the Western Blot Analysis (manufacturer’s data sheet). Negative controls were made omitting the primary antibody.

### Tissue Shrinkage

As nuclear volumes were obtained from areas measured in the Nissl-stained sections (see *Estimation of reference volume*), it was necessary to account for the possible shrinkage of tissue area during processing. To do this, tissue shrinkage was calculated from when the tissue blocks were immersed in sucrose until the sections were stained and ready to be observed at the microscope; note that the shrinkage occurring during fixation with paraformaldehyde could not be calculated because the AC blocks had been fixed by the donor institutions before they were sent to us. The area of the sections was measured in three steps during processing: 1. before and after the blocks were immersed in sucrose 30% (the anterior and posterior sides of each block were measured) (Fig. 1B), 2. when blocks were frozen on the microtome (one section every 0.6 mm was measured) (Fig. 1C), and 3. when sections were Nissl-stained, dehydrated and coverslipped (one section every 1.05 mm was measured) (Fig. 1D). Area was measured by superimposing a grid of points printed on a transparent sheet over the blocks or sections and counting the points that were on the tissue (Fig. 1). This analysis showed that the area did not change significantly in any of these processing steps (1% maximum difference); a finding that agrees with previous reports [21]–[23]. We also estimated the volume in three steps: 1. before and after the blocks of tissue were immersed in sucrose 30% (by liquid displacement), 2. during cutting, and 3. in dehydrated and coverslipped Nissl-stained sections. In the last two cases, the volume was calculated by using the above mentioned area measurements. The volume did not change significantly in any of the analyzed steps.

Despite the lack of area shrinkage, it is well known that frozen sections suffer a marked compression in the z-axis in the last steps of processing. In order to determine which step produces the most shrinkage, section thickness was measured with the microscope and the microcator using the newCAST software (see *Microscope setup)* just after the sections were cut on the freezing microtome and just after the sections were Nissl-stained, dehydrated and coverslipped. A thickness reduction of 8% after cutting on the microtome and a final thickness reduction of 70% were determined, concluding that most z-shrinkage occurred during the staining and/or drying steps. This reduction was taken into account in the estimation with the fractionator method of the cell numbers and did not affect the volume estimations.

### Delineation of the Amygdaloid Complex Nuclei

The different AC nuclei and their subdivisions were precisely delineated in every section included in the study following consistent criteria. Nissl-stained sections were photographed with a Nikon DXM1200F digital camera (Nikon, Tokyo, Japan) connected to the stereoscopic zoom microscope SMZ1500 (Nikon, Tokyo, Japan) at 0.75X; the boundaries of the AC nuclei were outlined over the printed Nissl images by observing the adjacent Nissl, AChE and Gallyas stained sections. For a more precise view of the stained sections we also used a Nikon Eclipse 80i microscope (Nikon, Japan).

**Figure 2:**
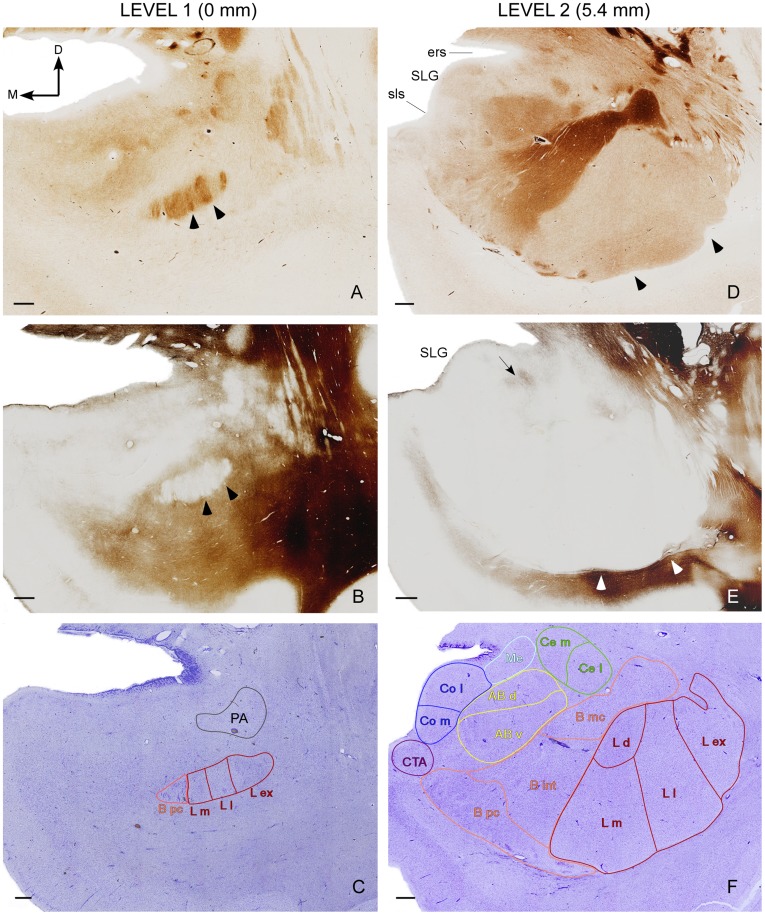
Delineation of the various subdivisions of the AC I. Series of adjacent coronal sections stained for acetylcholinesterase (A, D), Gallyas (B, E) and Nissl (C, F) in two anterior levels of the human AC (Levels 1 and 2). The distances from each level to the anterior commissure corresponding to the atlas of Mai et al. (2004) [26] are indicated on the top. The various AC nuclei and nuclear subdivisions shown in Table 2 have been outlined over Nissl sections. Arrowheads in A, B, D and E indicate the limits of the external, lateral and medial subdivisions of the lateral nucleus. The arrow in E indicates the limit between the accessory basal nucleus and the corticomedial group. Abbreviations: D, dorsal; ers, endorhinal sulcus; M, medial; SLG, semilunar gyrus; sls, semilunar (semiannular) sulcus. For other abbreviations see Table 2. Scale bar 1 mm.

**Figure 3:**
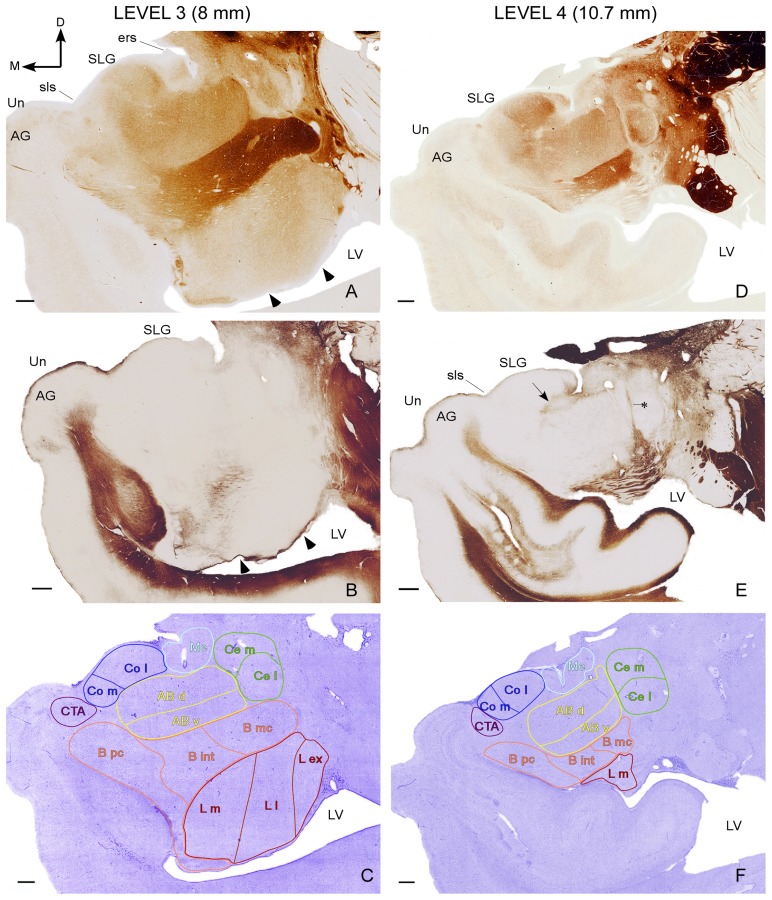
Delineation of the various subdivisions of the AC II. Series of adjacent coronal sections stained for acetylcholinesterase (A, D), Gallyas (B, E) and Nissl (C, F) in two posterior levels of the human AC (Levels 3 and 4). The distances from each level to the anterior commissure corresponding to the atlas of Mai et al. (2004) [26] are indicated on the top. The various AC nuclei and nuclear subdivisions shown in Table 2 have been outlined over Nissl sections. Arrowheads in A and B indicate the limits of the external, lateral and medial subdivisions of the lateral nucleus. The arrow in E indicates the limit between the accessory basal nucleus and the corticomedial group. The asterisk in E indicates the limit between the accessory basal and central nuclei. Abbreviations: AG, ambiens gyrus; D, dorsal; ers, endorhinal sulcus; LV, lateral ventricle; M, medial; SLG, semilunar gyrus; sls, semilunar (semiannular) sulcus; Un, uncus. For other abbreviations see Table 2. Scale bar 1 mm.

The divisions and nomenclature used in this study are very similar to that proposed by Sims and Williams [24] and also resemble those used by Schumann and Amaral [1] and Sorvari et al. [25] (Table 2). Four representative series of adjacent AChE, Nissl and Gallyas sections in the anteroposterior axis of the AC illustrate the delineation of the AC nuclei and their subdivisions observed in this study (Figs. 2 and 3).

**Table 2:** Divisions and nomenclature of the human AC.

NUCLEAR GROUPS	NUCLEI	NUCLEAR SUBDIVISIONS
**Basolateral group (BL)**	Lateral nucleus (L)	External (Lex)
		Lateral (Ll)
		Medial (Lm)
		Dorsal (Ld)
	Basal nucleus (B)	Magnocellular (Bmc)
		Intermediate (Bint)
		Parvocellular (Bpc)
	Accessory basal nucleus (AB)	Dorsal (ABd)
		Ventral (ABv)
**Corticomedial group (CM)**	Cortical nucleus (Co)	Medial (Com)
		Lateral (Col)
	Medial nucleus (Me)	–
**Central group (Ce)**	Ce	Medial (Cem)
		Lateral (Cel)
**Corticoamygdaloid transition area (CTA)**	CTA	–
**Periamygdalar area (PA)**	PA	–

AC, amygdaloid complex.

#### Periamygdalar area

The anterior amygdaloid area described by Sims and Williams [24] is the first structure that appeared at the most anterior AC level and remained in a dorsal and medial position to the lateral nucleus more caudally. As the boundaries of this region were rather fuzzy, especially in its medial part, only the most lateral region of the anterior amygdaloid area, which is called the periamygdalar area, was included here. The lateral limit of this area was defined by the first myelin bundle of the external capsule which separates it from the temporal claustrum following a ventromedial to dorsolateral direction (Fig. 2B); the remaining boundaries of the periamydalar area were defined by its lack of myelinated fibers and intense AChE staining, which stood out from the pale AChE activity of the surrounding anterior amygdaloid area (Fig. 2A–C). The periamydalar area disappears at midrostrocaudal levels of the AC. This region corresponds roughly to the preamygdalar claustrum described by Mai et al. [26].

#### Lateral nucleus

The lateral nucleus of the basolateral group was clearly defined in Nissl, Gallyas and AChE stained sections (Fig. 2A–C) as an oval-shaped structure that starts slightly posterior to the periamydalar area and is partially crossed by myelinated fibers. The AChE staining in the anterior level of the lateral nucleus is very intense (Fig. 2A), and it decreases considerably at mid and posterior levels (Figs. 2D, 3A). The boundary with the basal nucleus was defined easily at medium and posterior levels due to the heavy AChE staining of the latter (Figs. 2D, 3A). Nevertheless AChE staining strength is very similar in both nuclei at the anterior level, and the boundary between the basal and lateral nuclei was outlined by the higher density of Nissl-stained cells in the former compared to the latter.

The external subdivision of the lateral nucleus appears immediately anterior to the lateral and medial subdivisions, whereas the smallest dorsal subdivision occupies a short extension at midanteroposterior levels of the lateral nucleus (Figs. 2C, 2F, 3C, 3F). These subdivisions do not differ in the AChE stained sections, except at level 3 where the lateral subdivision shows a more intense AChE staining than the medial and external ones (Fig. 3A). Two little grooves at the ventral border between the external and lateral, and lateral and medial subdivisions (see arrowheads in Figs. 2A–B, 2D–E, 3A–B), together with blood vessels located among these subdivisions and several differences in the Nissl cellular density were used to trace longitudinal lines that delineated these three subdivisions from their ventral up to their dorsal borders. In Nissl sections, the external subdivision has medium to large darkly stained cells, the lateral subdivision has small-to-medium sized weakly stained cells, and the medial subdivision has small densely packed Nissl-stained cells (but not so packed as either the dorsal subdivision or the parvicellular subdivision of the basal nucleus). The dorsal subdivision of the lateral nucleus harbours more tightly packed and much smaller Nissl- stained cells than the other subdivisions, and it was located dorsal to the medial subdivision and medial to the most dorsal part of the lateral subdivision (Fig. 2F).

#### Basal nucleus

The basal nucleus appears slightly posterior and medial to the beginning of the lateral nucleus (Fig. 2A–C). Its heavy AChE staining was used to trace the boundaries of this nucleus with the accessory basal and the lateral nuclei at levels 2, 3 and 4. The delineation of the parvicellular, intermediate, and magnocellular subdivisions of this nucleus was made observing their AChE staining and the size of their Nissl-stained neurons, which increased considerably from the parvicellullar to the magnocellullar subdivisions. The parvicellular subdivision was the basal nucleus subdivision that appears at the most anterior level (Fig. 2C), followed by the intermediate and then the magnocellular subdivisions. The paralaminar region was included within the parvocellular subdivision because it was too vague and difficult to trace in all the sections.

#### Accessory basal nucleus

The accessory basal nucleus appears just posterior to the beginning of the lateral and basal nuclei, immediately medial to the basal nucleus from which it was distinguished by its low AChE activity (Fig. 2D, F). Its medial border was adjacent to the lateral border of both the cortical and medial nuclei of the corticomedial group. Apart from some differences in AChE staining, these two nuclei were separated by a small region that was lightly stained for Gallyas (see the black arrows in Figs. 2E, 3E). The boundary of the accessory basal nucleus with the central nucleus at levels 2–3 was defined by a lack (level 2) or a lower intensity (level 3) of AChE staining in the central nucleus compared to the accessory basal nucleus (Figs. 2D, 3A). At level 4, a thin Gallyas-positive strip demarcated the limit between both nuclei more sharply (see asterisk in Fig. 3E).

The dorsal and ventral subdivisions of the accessory basal nucleus were distinguished at levels 2–4 by the different sizes of their Nissl-stained neurons (Figs. 2F, 3C, 3F), as they are considered magnocellular and parvicellular, respectively. The AChE staining was slightly stronger in the dorsal than in the ventral subdivision, especially at level 4 of the AC (Fig. 3D).

#### Central nucleus

The central nucleus appears dorsal to the accessory basal nucleus at level 2 and it occupies the same region as the anterior amygdaloid area at more posterior levels (Fig. 2C, F); the transition of these two regions along the anteroposterior axis was defined by a Gallyas-positive staining located between the end of the anterior amygdaloid area and the beginning of the central nucleus (not shown). The AChE staining of the central nucleus is rather complex as it varies in its five different subdivisions along the anteroposterior axis [24]. At level 2 the central nucleus had no AChE, while the intensity of the staining increased at levels 3 and 4 (Figs. 2D, 3A, 3D). In this study, the central nucleus was exclusively divided in medial and lateral subdivisions that were distinguished because the former shows larger and more darkly Nissl-stained cells than the latter.

#### Cortical nucleus

The cortical nucleus is visible when the semilunar gyrus protrudes from the dorsomedial surface of the AC (Figs. 2F, 3C, 3F). The AChE staining of this nucleus was very low at level 2 and increased progressively at levels 3 and 4 (Figs. 2D, 3A, 3D).

Medial (ventral) and lateral (more dorsal) portions were distinguished based on differences in the AChE staining and in the density and distribution of the Nissl-stained neurons. The lateral subdivision shows a stronger AChE staining than the medial subdivision and its Nissl stained-neurons are slightly arranged in layers whereas those in the medial subdivision are more scattered (Figs. 2D–F, 3A–F).

#### Medial nucleus

The medial nucleus is located dorsolateral to the cortical nucleus, dorsomedial to the accessory basal nucleus and medioventral to the central nucleus (Figs. 2F, 3C, 3F). It could be distinguished from these nuclei by its lack or very low level of AChE staining and dorsally by the appearance of a region with strong myelin staining (Figs. 2D–E, 3A–B, 3D–E).

#### Corticoamygdaloid transition area

As described by Sims and Williams [24], a distinctive group of cells that separates the medial subdivision of the cortical nucleus from both the piriform cortex medially and from the parvicellular subdivision of the basal nucleus ventrolaterally appears underneath the semiannularis sulcus (Figs. 2F, 3C, 3F). Its light AChE staining was used to distinguish this area (Figs. 2D, 3A, 3D), although the Nissl-staining alone was sufficient to outline the region (Figs. 2F, 3C, 3F).

### Stereological Estimations

#### Microscope setup

An Olympus BX61 light microscope (Olympus, Tokyo, Japan) equipped with a microcator Heidenhahn MT 12 (0.5 µm resolution), an X-Y-Z motorized specimen stage (ProScan II, Prior Scientific, Cambridge, UK), and an Olympus DP-71 digital camera connected to a PC with two monitors that ran the newCAST stereology software package (Visiopharm, Hørsholm, Denmark) was used. The contours of the AC, its nuclei and nuclear subdivisions were drawn with the 1.25×PlanApo objective (Olympus, Tokio, Japan) following the delimitation criteria mentioned above.

#### Estimation of reference volume

The Cavalieri principle [27] was used to calculate the volume (V) of each AC nuclear group, nucleus and nuclear subdivision (equation 1: T (distance between sections)  = 1.05 mm; a (area per point)  = 0.59 mm^2^; ∑P, sum of points counted).
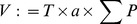
(1)


#### Estimation of cell numbers with the optical fractionator

The optical fractionator design [28], a combination of the optical disector [29] with a fractionator sampling scheme, was used to estimate the neuron, glia and endothelial cell numbers (*N*) in each AC nuclear group, nucleus and nuclear subdivision.

Thus, every region was systematically, uniformly and randomly sampled in the section plane (the x-y plane) and across the thickness of the sections (the z-axis), superimposing a grid of three-dimensional optical disectors over the microscope image displayed by the 100×UPlanSApo oil immersion objective (Olympus, Tokyo, Japan). In a previous pilot study, the proper sampling parameters to count at least 100 cells per studied region, which was enough to get a coefficient of error bellow 0.1, was established. Thus, the disector test frame area was 1760 µm^2^, and the step length, distance separating one disector from the next, ranged between 500–1000 µm, the same distance for both the x and the y axis, depending on the region size. As the mean section thickness was 13.6 µm, sampling in the z-axis was done with a disector height of 8 µm, keeping an upper guard zone of 3 µm.

The high variability in thickness measurements observed within a single section and between sections indicated that the z-axis compression was non-uniform; for this reason, we calculated the number weighted mean section thickness as 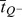 [30], [21]; this number reported information about whether many or few cells were situated in few/many thicker/thinner positions resulting in a more reliable number estimation. For that purpose, section thickness was measured at the center of the counting frame in one out of three disector containing at least one cell.

The number of each corresponding cell type was estimated with equation 2:
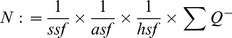
(2)where *ssf* is the section sampling fraction, *asf* the area sampling fraction, *hsf* the height sampling fraction and 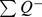
the number of cells counted in every region; *ssf* is calculated as 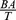, where *BA* is the block advance or thickness set at the microtome and *T* the distance between sections; *asf* is calculated as 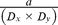, where *a* is the test frame area and *D_x_* and *D_y_* the step length in the x and y axis respectively; *hsf* is calculated as 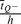
where 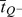
is the number-weighted mean section thickness (equation 3) and *h* the height of the disector
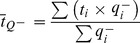
(3)in which ti is the local section thickness in the ith counting frame with a corresponding disector count of 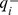.

#### Estimation of density

We estimated the density of neurons, glia and endothelial cells (*N_V_*) dividing the numbers obtained with the optical fractionator by the volume of each region as calculated by Cavalieri’s principle.
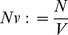
(4)


#### Precision of the stereological estimates

We calculated the coefficient of error (*CE*) due to the sampling method for both volume and neuron number estimates using the equations detailed in Gundersen and Jensen [27] and Gundersen et al. [31]. A minimum of 100 points for the Cavalieri estimations and 100 cells for the number estimations counted per region were enough to get a *CE* bellow 0.1. The mean *CE* for all cases 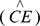 was obtained with equation 5 where *n* is the number of cases. Smaller regions such as the periamygdalar area or the corticoamygdaloid transition area or some small subdivisions of certain nuclei had higher *CEs* in the cell counts than the other AC regions, but increasing the number of sections or the sampling intensity in these small regions to achieve lower *CEs* would have required excessive microscope time.
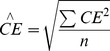
(5)


#### Statistical analysis

The correlation of the regional volumes, and the number and densities of every cell type with respect to the age at death of the subject that donated the sample was analyzed in every nuclei and nuclear subdivision using a Spearman's rho rank correlation test with the help of the SPSS statistical software. *P*-values below 0.05 were considered statistically significant, and those bellow 0.01, highly significant. Linear regression plots in the cases where the correlation was statistically significant were made with the GraphPad Prism software (version 5.0, CA, USA).

## Results

**Figure 4:**
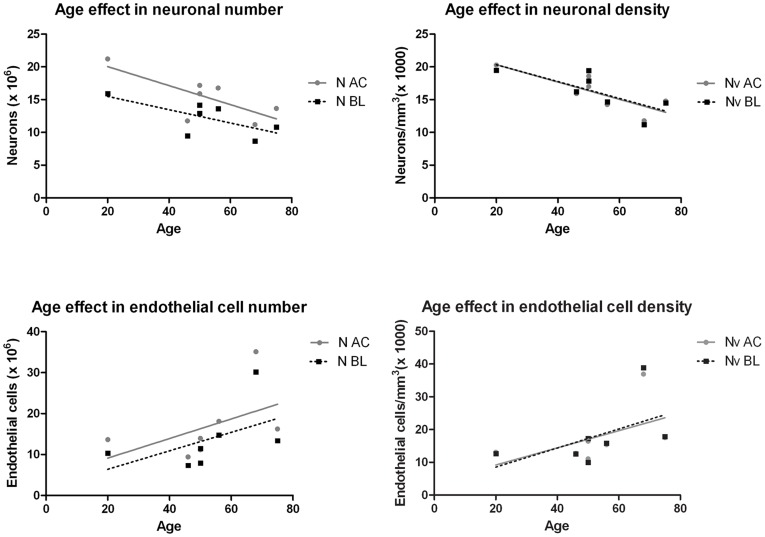
Age effect in the amount of neurons and endothelial cells. Linear regression plots of the number (N) and density (Nv) of neurons and endothelial cells as a function of the individuals’ age at death in the AC and in the BL.

The results of this study are summarized in Tables 3, 4, 5, 6 and Figure 4. With respect to the volume of the entire AC, its nuclear groups, nuclei and nuclear subdivisions (Table 3), our data show that the basolateral nuclear group occupies nearly 80% of AC volume, whereas the corticomedial and central nuclear groups together occupy only 16% of the volume. Within the huge basolateral nuclear group (volume of 759 mm^3^), the largest volume corresponded to the lateral nucleus, and volume decreased progressively from the basal to the accessory basal nuclei. The decrease was such that the volume of the accessory basal nucleus was nearly one third the volume of the lateral nucleus. In the case of the corticomedial nuclear group (volume of 94 mm^3^), nearly 70% of its volume is occupied by the cortical nucleus, with the lateral subdivision of the cortical nucleus being twice as large as its medial counterpart. The volume of the central nuclear group (60 mm^3^) was shared in nearly equal proportions between its medial and lateral subdivisions. The volume of either the total AC or any of its nuclear groups, nuclei or their subdivisions does not correlate with the subjects’ age at death (Table 6). The highest positive correlation was found in the accessory basal nucleus, although it did not reach statistical significance (Table 6).

**Table 3:** Volume of the AC, its nuclear groups, nuclei and nuclear subdivisions.

Neural structure	Volume (mm^3^)		
	Mean	SD	CE
**AC**	956	149	0.01
**BL**	759	111	0.02
**L**	376	68	0.01
**Lex**	97	24	0.02
**Ll**	128	16	0.02
**Lm**	132	24	0.03
**Ld**	24	7	0.06
**B**	259	40	0.02
**Bpc**	110	22	0.03
**Bint**	89	13	0.03
**Bmc**	60	10	0.04
**AB**	123	21	0.02
**Abd**	63	14	0.04
**Abv**	61	8	0.04
**CM**	94	17	0.05
**Co**	69	13	0.03
**Col**	49	9	0.04
**Com**	22	4	0.07
**Me**	28	5	0.05
**Ce**	60	8	0.04
**Cem**	30	6	0.05
**Cel**	29	4	0.05
**PA**	21	9	0.06
**CTA**	20	14	0.08

SD: standard deviation; CE: coefficient of error. For other abbreviations see Table 2.

**Table 4:** Number of neurons, glia and endothelial cells in the nuclear groups, nuclei and nuclear subdivisions of the AC.

Neural structure	N neurons (×10^6^)			N glia (×10^6^)			N endothelial cells (×10^6^)			N total cells (x 10^6^)		
	Mean	SD	CE	Mean	SD	CE	Mean	SD	CE	Mean	SD	CE
**AC**	15.39	3.50	0.03	60.01	12.04	0.02	16.81	8.58	0.03	92.20	17.64	0.03
**BL**	12.23	2.64	0.07	49.83	10.72	0.04	13.63	7.76	0.07	75.69	15.66	0.06
**L**	5.48	1.58	0.07	26.17	5.30	0.03	6.93	5.11	0.06	38.58	8.72	0.06
**Lex**	1.39	0.64	0.13	8.36	2.10	0.06	1.86	1.23	0.14	11.61	4.41	0.11
**Ll**	1.72	0.54	0.12	8.60	1.13	0.05	2.49	1.87	0.11	12.81	2.37	0.10
**Lm**	2.05	0.64	0.11	8.08	2.10	0.05	2.16	1.23	0.11	12.29	2.70	0.09
**Ld**	0.34	0.11	0.13	1.40	0.32	0.07	0.39	0.24	0.13	2.14	0.53	0.11
**B**	5.02	0.98	0.06	16.64	4.53	0.03	4.11	2.11	0.07	25.69	6.14	0.06
**Bpc**	3.04	0.52	0.08	6.51	1.82	0.05	1.45	0.66	0.12	10.99	2.43	0.09
**Bint**	1.30	0.36	0.12	5.80	1.88	0.06	1.48	0.93	0.13	8.58	2.52	0.11
**Bmc**	0.66	0.22	0.17	4.32	1.20	0.07	1.18	0.63	0.14	6.17	1.68	0.13
**AB**	1.73	0.39	0.09	7.02	1.62	0.05	2.49	0.90	0.08	11.24	2.21	0.07
**Abd**	0.83	0.15	0.13	3.69	1.09	0.06	1.30	0.54	0.11	5.83	1.39	0.11
**Abv**	0.90	0.28	0.13	3.33	0.68	0.07	1.19	0.47	0.12	5.42	1.10	0.11
**CM**	1.64	0.54	0.11	4.64	1.00	0.07	1.74	0.54	0.09	8.02	1.55	0.09
**Co**	1.14	0.43	0.10	3.18	0.64	0.06	1.37	0.39	0.09	5.70	1.09	0.08
**Col**	0.77	0.27	0.12	2.23	0.49	0.07	0.05	0.01	0.09	3.95	0.84	0.09
**Com**	0.37	0.17	0.17	0.96	0.21	0.10	0.04	0.01	0.17	1.74	0.36	0.15
**Me**	0.50	0.12	0.12	1.45	0.52	0.08	0.37	0.20	0.16	2.32	0.70	0.13
**Ce**	0.82	0.25	0.10	3.34	0.78	0.06	0.89	0.35	0.10	5.05	1.12	0.09
**Cem**	0.39	0.06	0.15	1.69	0.40	0.08	0.47	0.22	0.14	2.55	0.47	0.13
**Cel**	0.43	0.20	0.15	1.65	0.41	0.08	0.42	0.15	0.15	2.50	0.68	0.13
**PA**	0.37	0.22	0.16	1.46	0.51	0.08	0.28	0.19	0.20	2.11	0.86	0.16
**CTA**	0.33	0.14	0.17	0.72	0.22	0.12	0.28	0.13	0.19	1.32	0.38	0.16

N: number; SD: standard deviation; CE: coefficient of error. For other abbreviations see Table 2.

**Table 5:** Density of neurons, glia and endothelial cells in the nuclear groups, nuclei and nuclear subdivisions of the AC.

Neural structure	Nv neuron (×1000) (cells/mm3)		Nv glia (×1000) (cells/mm3)		Nv endotelial (×1000) (cells/mm3)		Nv total cells (×1000) (cells/mm3)	
	Mean	SD	Mean	SD	Mean	SD	Mean	SD
**AC**	16.09	2.84	62.79	8.85	17.60	8.82	96.80	13.33
**BL**	16.36	3.19	65.81	10.16	17.87	9.68	99.87	14.92
**L**	14.61	3.23	70.16	11.21	18.46	11.52	102.93	16.00
**Lex**	14.49	3.22	85.26	12.55	18.86	9.50	120.64	19.14
**Ll**	13.28	3.44	67.53	11.24	19.59	13.26	100.47	17.52
**Lm**	15.20	3.22	60.75	12.55	16.85	9.50	93.48	14.91
**Ld**	15.23	6.63	61.07	17.24	16.76	9.80	93.06	27.01
**B**	19.51	3.44	64.13	12.61	16.15	8.32	99.16	17.51
**Bpc**	28.20	4.49	59.26	12.03	13.35	5.85	100.54	14.26
**Bint**	14.62	3.47	64.96	16.83	17.01	10.23	96.21	23.52
**Bmc**	10.93	2.65	71.37	10.99	19.98	10.33	101.93	16.82
**AB**	13.77	3.50	56.73	7.81	20.16	7.46	91.07	11.49
**Abd**	13.65	3.51	58.68	9.87	21.07	9.08	93.40	14.10
**Abv**	14.80	4.02	54.75	7.27	19.41	7.19	88.95	11.99
**CM**	16.75	3.80	47.35	8.89	17.99	6.86	85.97	11.11
**Co**	16.21	4.06	45.41	5.47	19.73	6.09	82.24	8.21
**Col**	15.53	3.17	45.94	5.84	19.83	7.13	81.30	8.72
**Com**	16.99	4.81	44.73	7.82	19.08	6.21	80.80	9.84
**Me**	16.80	3.67	52.67	21.08	13.63	9.52	84.15	30.15
**Ce**	14.72	4.19	56.40	13.89	14.68	4.76	84.83	17.57
**Cem**	13.17	3.34	58.05	20.53	15.64	6.14	82.30	15.01
**Cel**	14.43	5.37	55.83	11.15	13.89	4.03	84.41	17.71
**PA**	16.73	4.32	81.03	17.81	14.09	7.16	106.07	29.20
**CTA**	17.79	7.25	40.60	15.22	16.19	11.24	77.96	22.73

Nv: number per volume (density); SD: standard deviation. For other abbreviations see Table 2.

**Table 6:** Coefficients of correlation (Spearman's rho test) of each variable studied with the subjects’ age at death for every AC nuclear group, nuclei and nuclear subdivision.

Neural Structure	Volume	N neurons	N glia	N endothelial	N total cells	Nv neurons	Nv glia	Nv endothelial	Nv total cells
**AC**	0.02	−0.51	0.38	0.76*	0.29	−0.78*	0.34	0.72	0.38
**BL**	0.02	−0.51	0.25	0.76*	0.38	−0.85*	0.31	0.72	0.38
**L**	0.05	−0.41	0.16	0.76*	0.38	−0.67	0.20	0.76*	0.47
**Lex**	0.05	−0.69	0.09	0.61	0.05	−0.76*	−0.11	0.65	0.18
**Ll**	−0.05	−0.56	0.69	0.96**	0.72	−0.65	0.34	0.85*	0.65
**Lm**	0.09	−0.27	−0.19	0.31	0.25	−0.60	0.09	0.14	0.04
**Ld**	0.37	−0.29	0.58	0.78*	0.36	−0.43	0.04	0.60	0.04
**B**	0.23	−0.45	0.18	0.70	0.32	−0.51	0.16	0.76*	0.38
**Bpc**	0.14	0.32	0.74	0.83*	0.61	−0.38	0.27	0.87*	0.63
**Bint**	0.02	−0.41	0.14	0.36	0.32	−0.69	0.31	0.38	0.16
**Bmc**	−0.02	−0.45	−0.13	0.70	0.05	−0.47	−0.02	0.81*	0.22
**AB**	0.49	−0.29	0.58	0.29	0.63	−0.69	0.20	0.04	−0.09
**Abd**	0.67	−0.25	0.78*	0.32	0.81*	−0.72	−0.02	−0.05	−0.05
**Abv**	0.02	−0.36	0.41	0.60	0.45	−0.70	0.20	0.49	0.27
**CM**	0.05	−0.54	0.32	0.32	0.23	−0.14	0.38	0.76*	0.16
**Co**	−0.12	−0.18	0.23	0.32	0.23	−0.43	0.56	0.36	0.20
**Col**	−0.35	−0.32	−0.18	−0.04	−0.20	−0.22	0.67	0.00	0.38
**Com**	−0.18	−0.07	0.11	0.58	0.20	−0.63	0.38	0.60	0.38
**Me**	0.22	−0.20	0.40	0.70	0.58	−0.34	0.27	0.47	0.27
**Ce**	0.16	−0.49	−0.02	−0.13	−0.23	−0.56	−0.27	−0.45	−0.56
**Cem**	0.16	−0.14	−0.11	−0.05	0.04	−0.51	−0.36	−0.27	−0.51
**Cel**	−0.45	−0.49	−0.11	−0.41	−0.29	−0.63	−0.14	−0.40	−0.18
**PA**	−0.35	−0.31	0.02	0.41	0.02	−0.07	0.69	0.74	0.65
**CTA**	−0.09	−0.20	0.05	0.69	−0.04	−0.25	0.52	0.70	0.36

N, number; Nv, density. For other abbreviations see Table 2.

* Significant (p<0.05) and

** highly significant (p<0.01) correlation coefficients.

The mean, standard deviation and coefficient of error for the numbers of neurons, glia, endothelial cells and total cells in the whole AC, its nuclear groups, nuclei and nuclear subdivisions are summarized in Table 4. Our data show that the mean amounts of neurons, glia and endothelial cells (x10^6^) in the entire AC are 15.39±3.50, 60.01±12.04, and 16.81±8.58, respectively. The number of neurons is about one quarter the amount of glia cells in the entire AC and in its nuclear groups, so there are four times more glia cells than neurons in the AC. The number of endothelial cells is very similar to that of neurons in every region of the AC examined. Against the total cell population of the AC, the percentages of neurons, glia and endothelial cells are about 16%, 65% and 17%, respectively, and these percentages are roughly maintained in each AC nuclear group, nucleus and nuclear subdivision. As shown in Table 6, the neuron number showed high and moderate negative coefficients of correlation with respect to the subject’s age at death in every AC region except the parvocellular subdivision of the basal nucleus, which showed a positive correlation coefficient, although none of these correlations reached statistical significance. Glia number showed moderate positive coefficients of correlation with age in most AC territories, and in the dorsal subdivision of the accessory basal nucleus this correlation was statistically significant (Table 6). Endothelial cell number showed high positive correlation coefficients in most of the AC regions, and in the whole AC, the basolateral group, lateral nucleus (and specifically its lateral and dorsal subdivisions) and in the parvocellular subdivision of the basal nucleus the correlations were statistically significant (Table 6). These data indicate that the number of endothelial cells increases substantially in the AC during aging, and the amounts of neurons and glia show a respective moderate tendency to decrease and increase. The coefficients of correlation between total cell number and age at death in most of the AC regions were positive and in the dorsal subdivision of the accessory basal nuclei this relation was statistically significant (p<0.05) (Table 6).

The mean, standard deviation and coefficient of error of the density of neurons, glia, endothelial cells and total cells in the whole AC, its nuclear groups, nuclei and nuclear subdivisions are summarized in Table 5. The mean density of neurons, glia and endothelial cells (x10^3^) in the entire AC are 16.09±2.84, 62.79±8.85 and 17.60±8.82, respectively. The density of neurons is fairly uniform throughout the AC, except for the parvocellular subdivision of the basal nucleus which has a very high neuron density due to the small size of its neurons. AC endothelial cell density is quite homogeneous but the density of glial cells increases notably in lateral AC regions such as the external subdivision of the lateral nucleus and the periamygdalar area. This increase in glial density is probably due to the high number of myelinated fiber bundles that traverse these areas of the AC.

Neuron density showed statistically significant negative correlations with the subject’s age at death in the entire AC, the basolateral group and in the external subdivision of the lateral nucleus; almost significant negative correlation coefficients were found in the rest of AC regions. Glial density did not reach any statistically significant correlation. In contrast, endothelial cell density showed highly positive correlation coefficients in most of the AC regions and these positive correlations reached statistical significance in the lateral nucleus (lateral subdivision), basal nucleus (parvocellular and magnocellular subdivisions) and the corticomedial group of the AC. Finally, the coefficients of correlation between total cell density and the subject’s age at death were positive in every AC territory except in the central nucleus but none of them reached statistical significance.

The age-trends observed for the amount of neurons and endothelial cells were maintained when the analysis of the correlation were performed omitting, respectively, the 20- and 68-year old subjects from the data analysis (Figure S1), which were the cases whose extreme data could have driven these variations as seen in Figure 4.

## Discussion

A detailed analysis of the volume and cell numbers and densities of individual nuclei in the amygdala of control subjects is needed to enable comparison with psychiatric diseases that might produce structural changes exclusively in specific nuclei. This study reports quantitative data on the regional volume, and cell number and density determined with unbiased stereological techniques in the whole AC, and in each of its nuclear groups, including their nuclei and their nuclear subdivisions; all were carefully delineated following the guidelines provided by previous detailed and widely used anatomical studies. Besides the whole AC, we have examined the 5 AC nuclear groups, 5 nuclei and 13 nuclear subdivisions. First of all, the volume of these 24 AC territories was estimated and the boundaries exactly delineated as described so as to be used in further studies. Next, we counted the total numbers of neurons, glia and endothelial cells, and these data together with their densities are provided for each of the 24 territories. To our knowledge this is the first study that provides the total number and density of cells in the AC as a whole and in its various subdivisions in the same samples of human tissue. Finally, we also determined the coefficients of correlation between all the variables analyzed here and the ages at the time of death of the subjects that had donated the tissue.

### Methodological Considerations

Volume and density estimations should be analyzed carefully given the shrinkage and/or swelling that may occur between the subject’s death and the time at which the estimations are made. Quantitative studies should control and correct for this shrinkage so as to guarantee that the obtained data are accurate and allow valid comparisons. In the present study, we demonstrate that freezing “embedding" has no effect on the area shrinkage and that z-axis shrinkage occurred mainly in the last steps of dehydration. Thus, the estimations of volume performed here could have only been affected by the shrinkage occurring during paraformaldehyde fixation (see *Tissue shrinkage* in *Materials and Methods*). Although this step of tissue processing is considered to not be the principal cause of shrinkage [32] it has been reported to produce about 2.7% shrinkage [33]. The cell number estimations using an optical fractionator design are not affected by tissue shrinkage, and therefore the density assessments calculated dividing the cell number by volume would have only been affected by the same shrinkage as the volume estimations.

In their study, Gardella et al. [34] demonstrated that the density of particles through the thickness of frozen sections was fairly uniform, indicating that there is an absence of differential distortion through the z-axis. Nevertheless these authors observed a trend towards a slight increase in the density of particles at the mid portions of the thickness of the sections, probably due to a smoother compression at the uppermost and lowermost surfaces with regard to the center and/or to some loss of particles in these surfaces, what is known as the “lost caps effect" [35]. The work of Berreta et al. [2] showed that the total numbers of cells estimated in several nuclei of the AC without keeping guard areas were on average 25–27% lower than the numbers obtained in an optical disector design that respected guard areas. In order to analyze the distribution of cells throughout the disector height, and also with the aim of assuring that the upper and lower guard areas were large enough to avoid the lost cap effect, in three cases included in this study we computed the z-axis coordinate of up to 5422 cells and checked that their distribution along the disector thickness was sufficiently homogeneous (Fig. S2). In one section of one of the subjects, we also obtained the z-coordinate for 400 cells along the complete thickness of the section, observing that the number of cells decreased from the center towards the periphery of the section thickness, and that this decrease occurred outside of the established disector height (Fig. S2). These observations agree with the calibration study described by Dorph-Petersen et al. [36] and indicate that the distribution of cells in the z-axis might show a higher degree of variation than previously reported by Gardella et al. [34].

We used the nucleolus instead of the nucleus as a counting unit for neurons when using the optical disector probe when it was possible as the smaller size of the nucleolus compared to the nucleus better fulfills the two requirements of the optical disector probe: that each cell can be counted only once and that any cell has the same probability of being counted as any other [37]. Moreover, instead of the common mean thickness we calculated the number-weighted mean section thickness, a value that weights the thickness measurement with respect to the cell number counted in each position where the thickness is measured and gives a more reliable number estimation when section thickness is compressed in a non-uniform pattern, as happens in the last steps of freezing procedures [21], [30].

This study has been performed using human samples with low post mortem intervals, which is considered to ensure high tissue quality [38]. It is noteworthy, however, that the control cases included here had different causes of death (Table 1) and some of them, especially those suffering cardiac problems or carcinoma, may have influenced the amount of cell number, especially the endothelial cells. Another critical point when estimating the total number of cells in subdivisions of the AC with the optical fractionator is to establish a clear definition of subdivision boundaries. In this aspect we have roughly followed the boundaries proposed by Sims and Williams [24] and Schumann and Amaral [1] with some differences that were intended to avoid ambiguous nuclear delimitations.

### Volume

The volume of the human AC is approximately 950 mm^3^ with more than 80% corresponding to the basolateral group, 10% to the corticomedial group and 6% to the central group. Overall, the volumes of the AC nuclei obtained in the present study fall within the ranges reported previously by other authors [2], [5], [6], [7], [39], [40]. In the case of the volume of the total AC, the value estimated in this study is smaller than that reported by Schumann and Amaral [7]. This difference can be explained by structures such as the nucleus of the lateral olfactory tract, the anterior amygdaloid area, the periamygdaloid cortex and the amygdalohippocampal area, which have not been included in this study due to the difficulties of precisely and objectively outlining their boundaries, being included by these other authors as within the AC. However, the volumes of the individual AC nuclei estimated by the same authors were more similar to those reported here, although still a bit higher especially in the lateral and basal nuclei. Other studies have made smaller volume estimations than ours [2], [39] most probably due to differential shrinkage suffered during the tissue processing or to a different delineation of the nuclei. In the case of Chance et al. [39] the differences might be because they used paraffin as an embedding medium, and this medium is known to cause shrinkage of about 50% in volume [21]. In the case of Berreta et al. [2], the differences could be related to the fact that the human tissue they used was immersed in paraformaldehyde for three weeks and may therefore have suffered a greater degree of shrinkage.

Determination of the volume of the AC has been the target of many studies on psychiatric disorders [2], [5], [6], [39], [40], [41]. We hope the reliable volume estimations we provide for the entire AC and of each of its nuclear groups and nuclei from individuals without any neurological or psychiatric disease can be useful for future studies to detect variations in the size of certain AC territories that might occur in pathologies such as bipolar disorder or schizophrenia.

### Number and Density of Neurons, Glia and Endothelial Cells

There are approximately 15 million neurons in the human AC with nearly 80% residing in the basolateral group, 10% in the corticomedial group and 5% in the central group. The number of endothelial cells is rather similar to that of neurons whereas the number of glia is approximately four times higher than neurons. This glia-neuron ratio differs from that reported in the human cerebral cortex, where it ranges from 1.55 to 2.19 depending on the cortical area examined [47], and in subcortical structures, which vary from 14 in the mediodorsal thalamic nucleus and ventral pallidum to 3 in the nucleus accumbens [6].

Previous studies have given quantitative measurements for neurons or glia cells in the AC but not many have provided data for both neurons and glia and none but this one has given data for all three types: neurons, glia and endothelial cells. In terms of neuron number, our estimations are comparable, although slightly higher, to those reported by Schuman and Amaral [1] and Kreczmanski et al. [5] in the lateral, basal, accessory basal and central nuclei, which are the AC nuclei analyzed in their studies. However, in these same nuclei Berreta et al. [2] reported considerably lower neuronal numbers. This variation in the amount of neurons is likely to be the result of a different delineation of the AC nuclei, which would also explain the smaller volumes of the AC nuclei reported by these authors compared to ours. An important procedural aspect that might explain the variations in the amount of neurons found in this and previous studies is that we have calculated the mean thickness for each section taking into account the number of neurons in each position where thickness was measured (i.e. the number weighted mean section thickness) [21], [30], which gives a more reliable number estimation when section thickness is compressed in a non-uniform pattern.

In terms of neuron density, our estimations are quite similar to those reported by Bowley et al. [4] for the whole AC or Kreczmanski et al. [5] in the lateral nucleus. However, neuron densities estimated by Schuman and Amaral [7] or Berreta et al. [2] for the whole AC and for some of its nuclei were lower than the ones we have obtained. In the case of Schuman and Amaral [7] these differences were to be expected since, as previously mentioned, they reported slightly lower neuron numbers and higher volumes than our study. The Berreta et al. study [2] showed both lower neuron numbers and smaller volumes than our study, and it is difficult to explain the reasons for this different neuronal density. The differences in neuron density that we observed among the AC nuclei were consistent with the data reported by all of these studies except that the highest neuronal density in our study was found in the basal nucleus and in the rest of the studies it was in the cortical or the central nuclei. Such a high neuronal density in the basal nucleus is due to the very high neuronal density of its parvocellullar subdivision, which is twice the density of the rest of the AC nuclei, and to our including in this parvocellular subdivision the paralaminar area, which contains a pool of immature neurons in the adult brain [42], [43], [44].

Recently, the study of Carlo et al. [45] analyzed the neuron number and the volume of different AC nuclei and nuclear subdivisions in several non-human primate species. Although their numbers and volumes are markedly lower than the ones obtained for the human brain in this study, the percentages among nuclei and their subdivisions are roughly similar. These authors found that across primate species including humans the volume and neuron number of the central nucleus increase more slowly than those in the rest of AC nuclei.

Numbers and densities of glia cells obtained in the present study are consistent with previous reports [4], [8]. The high glial density found in the lateral nucleus may be related to the numerous myelinated fibers that traverse this nucleus, probably because it is the main input for sensory association cortical connections [46]. Thus, as oligodendrocytes are supposed to be the glial cell type that suffers a reduction in number in some psychiatric disorders [8], the lateral nucleus could be specifically affected just as its neuronal density, nuclear volume and neuron size are affected [2], [5], [7].

Our data indicate that the number of endothelial cells is similar to that of the neurons in the whole AC in every of its nuclei and nuclear subdivisions. We also observed an increase in the density of endothelial cells in some AC nuclei with advancing age, as explained below. As yet, no other studies have focused on determining the number or density of endothelial cells in the AC in either controls or pathological conditions. In the monkey parietal cortex, Konopaske et al. [20] estimated 25,000 endothelial cells/mm^3^ and this density was found to increase in antipsychotic-exposed monkeys.

### The Aging Effect

The AC is involved in Alzheimeŕs disease as well as in healthy age-related cognitive and memory deficits. Thus, understanding the changes that might occur in this subcortical structure during normal aging is crucial to facilitate diagnosis in early stages of Alzheimeŕs disease [48], [49]. The present study has shown that the amount of AC neurons and endothelial cells displays a tendency to decrease or increase with aging, respectively, and this effect was found to reach statistical significance in the AC and in its basolateral group. The coefficients of correlation for the number and density of cells with respect to the subject’s age at death in the other AC nuclei were still high but not significant, a finding that might be attributed to a low power of the statistical analysis due to the lack of tissue at certain ages. These age effect trends need to be confirmed in future aging studies.

Neuroimaging studies have indicated a grey matter volume decline in the AC during aging [50], [51]. In our study, changes in neither the volume of the AC nor that of any of its various subdivisions showed significant correlation with age. The volume of the accessory basal nucleus, specifically its dorsal subdivision, nevertheless did show a high positive correlation coefficient with age that is likely to be related to the significant positive correlation of age with glia number but not density, in this AC nuclear subdivision.

Nearly all the AC subdivisions showed high negative correlation coefficients for neuron number and age, indicating a tendency toward age-dependent neuronal loss, although none of them reached statistical significance. Neuronal density also decreases with subjects’ age at death and this decrease reached statistical significance in large territories such as the AC and its basolateral group. However, as density is a secondary parameter (ratio of absolute number and volume), and the AC or the basolateral group showed null correlation with the subjects’ ages for volume and no statistically significant correlations for neuron numbers, the significance showed in density could be due more to the error propagation than to a real significant decrease.

Pakkenberg and Gundersen [11] demonstrated a 10% reduction in the neuron number of the neocortex in aged subjects. In the same subjects, they also found a white matter volume reduction which they thought was related to a degeneration of myelinated fibers and which could be responsible for brain function loss with advancing age. Our results indicate that the number and density of glial cells in the grey matter of AC nuclei tend to increase moderately with aging, which could be a compensatory mechanism or a glial cell population response to neuronal loss. Both gliosis and fiber loss have been described as stages of age-dependent degeneration [52] and similar mechanisms occur in neurodegenerative diseases in which astrocytes are increased in size and number [53], [54], [55], [56].

It is interesting to note that the parvocellular subdivision of the basal nucleus was the only AC region that did not show a negative correlation coefficient between neuron number and age. This result can be explained by the fact that this subdivision of the basal nucleus includes the paralaminar region, which contains a pool of immature neurons in the adult brain that might counteract the decrease in neuron number [43], [44].

Endothelial cell number increased with age in nearly all the subdivisions of the AC, but the correlation was statistically significant only in the whole AC and the basolateral group. In terms of endothelial density, the correlation was higher in the AC and in the basolateral and corticomedial groups. It has been found that cerebral blood flow decreases in the AC with increasing age [57] and that chronic exposure to hypoxic environments can lead to structural and functional adaptations of the brain microvasculature, including an increase in capillary density [58]. These studies support the hypothesis that the increase of the endothelial cell number and density in the AC and in most of its nuclei during aging is a brain adaptation to counteract the age-related blood flow decrease with the aim of maintaining oxygen delivery to these regions. It is also known that these adaptation mechanisms decrease with age [58], and might not be efficient enough to counteract the neuronal loss observed during aging in the present study.

## Supporting Information

Figure S1 **Age effect in the amount of neurons and endothelial cells removing the cases 1 and 5 (** **Table 1** **).** Linear regression plots of the number (N) and density (Nv) of neurons and endothelial cells as a function of the individuals’ age at death in the AC and in the BL of six cases of the study (omitting the 20 years old case for neuronal number and density and the 68 years old case for the endothelial cell number and density).
(TIF)

Figure S2 **Distribution of cells within the section thickness. A.** Calibration study to determine the distribution of cells (y axis) in each z position (x axis) within the full section thickness of one section of the AC from one of the subjects included in this study. A total of 400 cells were counted in the AC contained in this section. **B.** Calibration study within the optical disector thickness obtained after Nissl-stained counting of a total of 5422 cells of the AC in the complete set of sections from three subjects included in this study.
(TIF)

Table S1 Number of neurons obtained with Nissl and NeuN stainings.
(DOCX)
