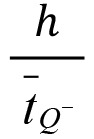# Correction: Stereological Analysis of Neuron, Glial and Endothelial Cell Numbers in the Human Amygdaloid Complex

**DOI:** 10.1371/annotation/42d5754a-295b-466e-ae55-6f9f41b06760

**Published:** 2012-11-06

**Authors:** María García-Amado, Lucía Prensa

There is an error in the equation. The correct equation is displayed here: