# Supplementary figures and images for: Stereological Analysis of Neuron, Glial and Endothelial Cell Numbers in the Human Amygdaloid Complex
Source: PLoS One. 2012 Jun 13;7(6):e38692. doi: 10.1371/journal.pone.0038692 (PMC3374818; doi:10.1371/journal.pone.0038692)

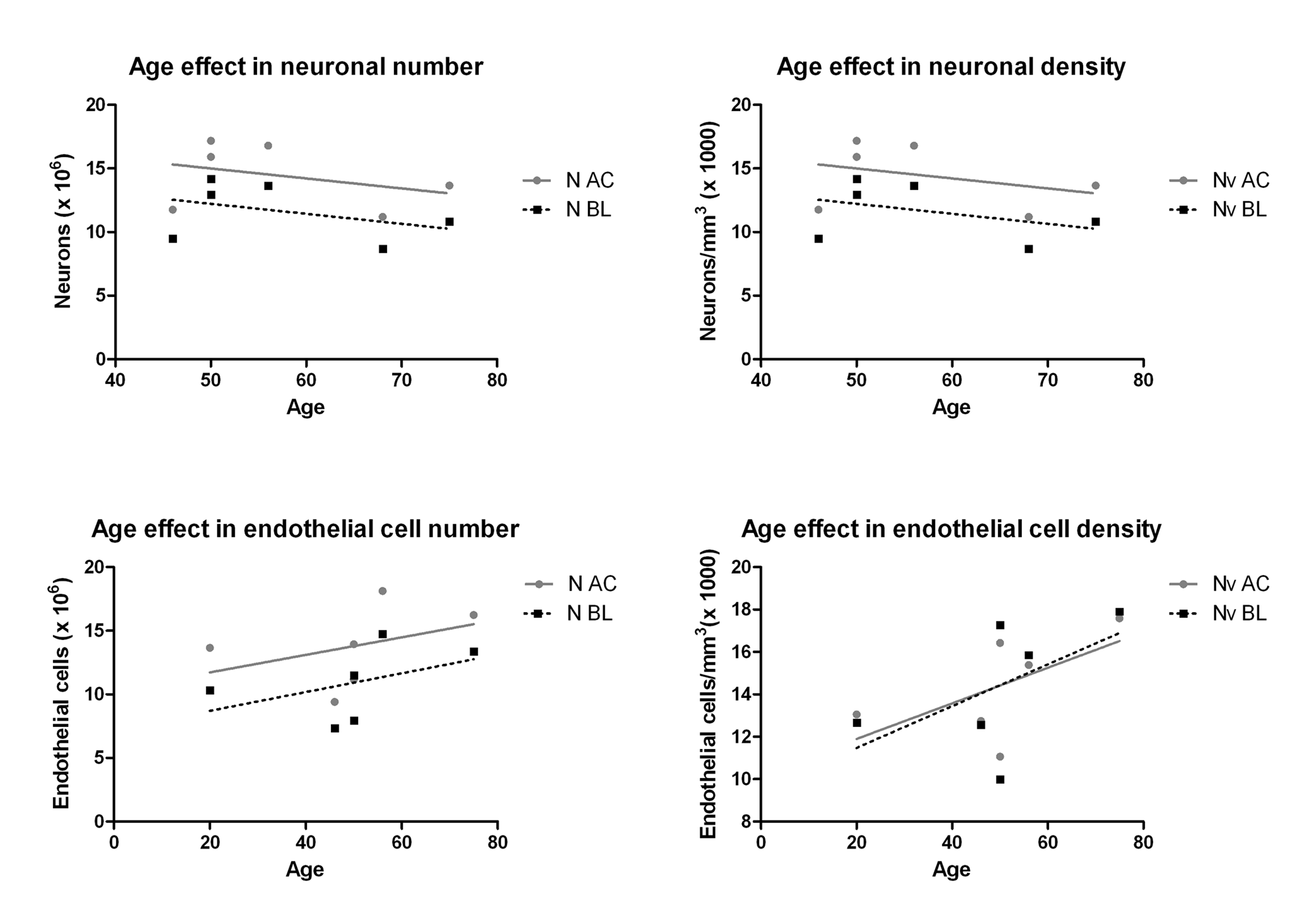

Supplement: Figure S1 — Age effect in the amount of neurons and endothelial cells removing the cases 1 and 5 ( Table 1 ). Linear regression plots of the number (N) and density (Nv) of neurons and endothelial cells as a function of the individuals’ age at death in the AC and in the BL of six cases of the study (omitting the 20 years old case for neuronal number and density and the 68 years old case for the endothelial cell number and density). (TIF) [file pone.0038692.s001.tif]

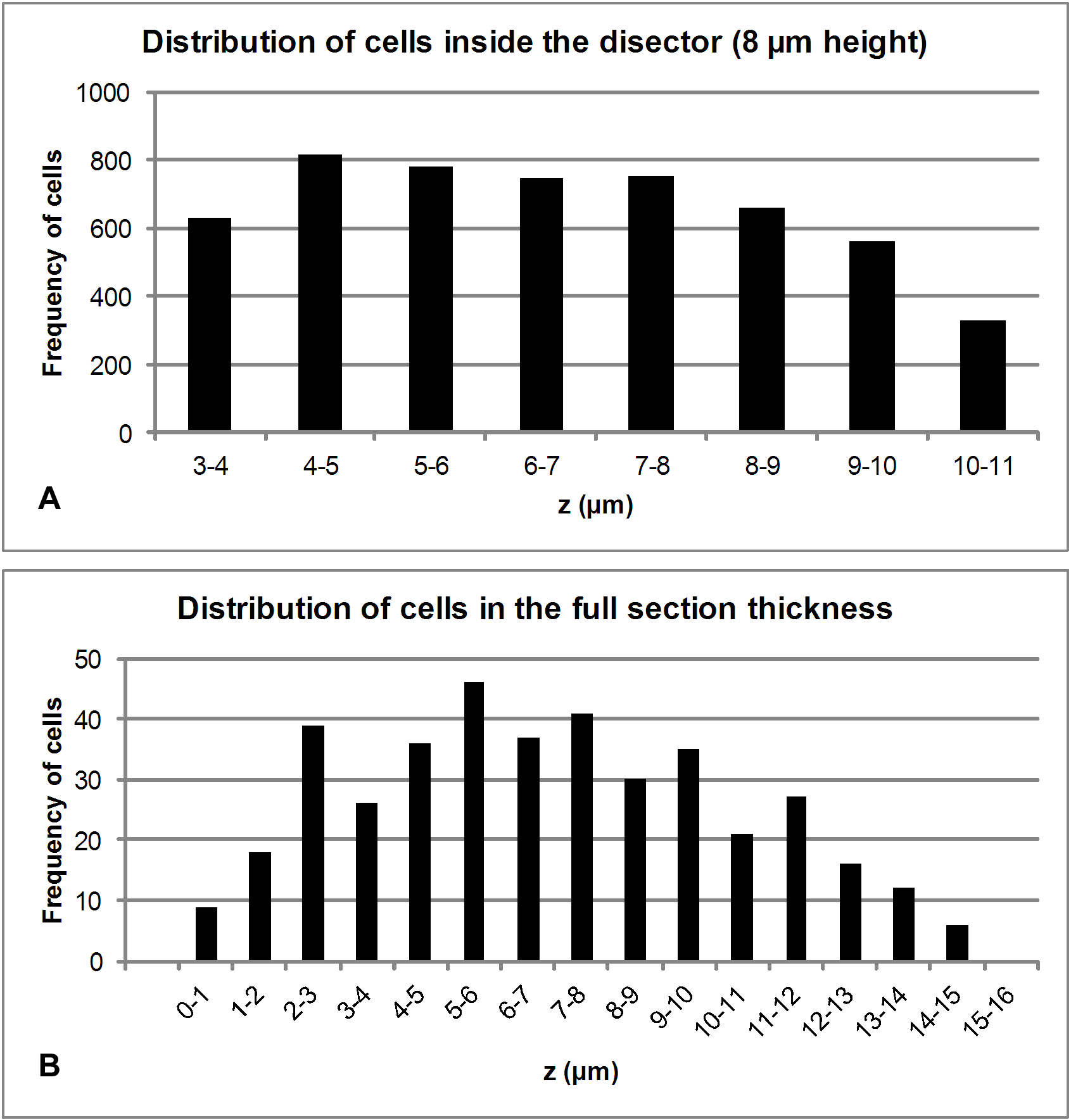

Supplement: Figure S2 — Distribution of cells within the section thickness. A. Calibration study to determine the distribution of cells (y axis) in each z position (x axis) within the full section thickness of one section of the AC from one of the subjects included in this study. A total of 400 cells were counted in the AC contained in this section. B. Calibration study within the optical disector thickness obtained after Nissl-stained counting of a total of 5422 cells of the AC in the complete set of sections from three subjects included in this study. (TIF) [file pone.0038692.s002.tif]
