# Supplementary material for: Stereological Analysis of Neuron, Glial and Endothelial Cell Numbers in the Human Amygdaloid Complex
Source: PLoS One. 2012 Jun 13;7(6):e38692. doi: 10.1371/journal.pone.0038692 (PMC3374818; doi:10.1371/journal.pone.0038692)
Supplement: Table S1 — Number of neurons obtained with Nissl and NeuN stainings. (DOCX) [file pone.0038692.s003.docx]

**Table 1S.** Number of neurons obtained with Nissl and NeuN stainings

| **Neural structure** | **N neurons (CE)** | | **Nissl-NeuN difference (%)** |
| --- | --- | --- | --- |
|  | **Nissl** | **NeuN** |  |
| **AC** | 11.502.303 (0.03) | 11.366.732 (0.03) | + 1 % |
| **BL** | 9.555.073 (0.08) | 9.430.883 (0.07) | + 1 % |
| **L** | 4.027.785 (0.08) | 4.091.742 (0.07) | -2 % |
| **B** | 4.116.402 (0.07) | 3.743.678 (0.05) | + 9 % |
| **AB** | 1.410.886 (0.08) | 1.595.463 (0.08) | - 12 % |
| **CM** | 1.203.231 (0.1) | 1.236.203 (0.09) | - 3% |
| **Co** | 802.531 (0.1) | 734.006 (0.1) | + 9 % |
| **Me** | 445.169 (0.1) | 419.653 (0.08) | + 6 % |
| **Ce** | 743.999 (0.1) | 699.646 (0.08) | + 6 % |

N: number; CE: coefficient of error. For other abbreviations see Table 2
